# Common and Distinct Clinical Features in Adult Patients with Anti-Aminoacyl-tRNA Synthetase Antibodies: Heterogeneity within the Syndrome

**DOI:** 10.1371/journal.pone.0060442

**Published:** 2013-04-03

**Authors:** Yasuhito Hamaguchi, Manabu Fujimoto, Takashi Matsushita, Kenzo Kaji, Kazuhiro Komura, Minoru Hasegawa, Masanari Kodera, Eiji Muroi, Keita Fujikawa, Mariko Seishima, Hidehiro Yamada, Ryo Yamada, Shinichi Sato, Kazuhiko Takehara, Masataka Kuwana

**Affiliations:** 1 Department of Dermatology, Kanazawa University Graduate School of Medical Science, Kanazawa, Japan; 2 Department of Dermatology, Social Insurance Chukyo Hospital, Nagoya, Japan; 3 Department of Dermatology, Nagasaki University Graduate School of Biomedical Sciences, Nagasaki, Japan; 4 Unit of Translational Medicine, Department of Immunology and Rheumatology, Nagasaki University Graduate School of Biomedical Sciences, Nagasaki, Japan; 5 Department of Dermatology, Ogaki Municipal Hospital, Ogaki, Japan; 6 Division of Rheumatology, Department of Internal Medicine, and Allergy, St. Marianna University, Kawasaki, Japan; 7 Center for Genomic Medicine, Graduate School of Medicine, Kyoto University, Kyoto, Japan; 8 Department of Dermatology, Faculty of Medicine, University of Tokyo, Tokyo, Japan; 9 Division of Rheumatology, Department of Internal Medicine, Keio University School of Medicine, Tokyo, Japan; National Institutes of Health, United States of America

## Abstract

**Objective:**

To identify similarities and differences in the clinical features of adult Japanese patients with individual anti-aminoacyl-tRNA synthetase antibodies (anti-ARS Abs).

**Methods:**

This was a retrospective analysis of 166 adult Japanese patients with anti-ARS Abs detected by immunoprecipitation assays. These patients had visited Kanazawa University Hospital or collaborating medical centers from 2003 to 2009.

**Results:**

Anti-ARS Ab specificity included anti-Jo-1 (36%), anti-EJ (23%), anti-PL-7 (18%), anti-PL-12 (11%), anti-KS (8%), and anti-OJ (5%). These anti-ARS Abs were mutually exclusive, except for one serum Ab that had both anti-PL-7 and PL-12 reactivity. Myositis was closely associated with anti-Jo-1, anti-EJ, and anti-PL-7, while interstitial lung disease (ILD) was correlated with all 6 anti-ARS Abs. Dermatomyositis (DM)-specific skin manifestations (heliotrope rash and Gottron’s sign) were frequently observed in patients with anti-Jo-1, anti-EJ, anti-PL-7, and anti-PL-12. Therefore, most clinical diagnoses were polymyositis or DM for anti-Jo-1, anti-EJ, and anti-PL-7; clinically amyopathic DM or ILD for anti-PL-12; and ILD for anti-KS and anti-OJ. Patients with anti-Jo-1, anti-EJ, and anti-PL-7 developed myositis later if they had ILD alone at the time of disease onset, and most patients with anti-ARS Abs eventually developed ILD if they did not have ILD at disease onset.

**Conclusion:**

Patients with anti-ARS Abs are relatively homogeneous. However, the distribution and timing of myositis, ILD, and rashes differ among patients with individual anti-ARS Abs. Thus, identification of individual anti-ARS Abs is beneficial to define this rather homogeneous subset and to predict clinical outcomes within the “anti-synthetase syndrome.”

## Introduction

The presence of autoantibodies (Abs) is one of the hallmarks of connective tissue diseases, such as systemic lupus erythematosus (SLE), systemic sclerosis (SSc), and idiopathic inflammatory myopathy. In particular, a variety of serum Abs is found in patients with idiopathic inflammatory myopathies, including polymyositis (PM) and dermatomyositis (DM) [1], [2]. It is clinically of considerable importance to identify Abs in patients with PM/DM, because each Ab is closely associated with certain clinical features [3]. For example, anti-Mi-2 is associated with classic DM without interstitial lung disease (ILD) or malignancy and with good response to treatment [4]–[6]; anti-155/140 is associated with malignancy-associated or juvenile DM [7]–[10]; and anti-CADM-140/MDA5 is associated with clinically amyopathic DM (CADM) and rapidly progressive-ILD (RP-ILD) that results in poor prognosis [11], [12]. Abs reactive with aminoacyl-tRNA synthetases (ARS) are also representative Abs that are detected in patients with PM/DM. Eight anti-ARS Abs have been described: anti-histidyl (anti-Jo-1), anti-threonyl (anti-PL-7), anti-alanyl (anti-PL-12), anti-glycyl (anti-EJ), anti-isoleucyl (anti-OJ), anti-asparaginyl (anti-KS), anti-phenylalanyl (anti-Zo), and anti-tyrosyl (anti-Ha) tRNAs [13]–[20]. Based on a unique combination of clinical features commonly observed in patients with anti-ARS Abs, Targoff proposed a disease entity termed “anti-synthetase syndrome,” which is characterized by myositis, ILD, fever, Raynaud’s phenomenon, arthritis, and mechanic’s hands [21]. Although anti-synthetase syndrome has common clinical manifestations, further observations have distinguished some differences in clinical features associated with individual anti-ARS Abs [22]. For example, it has been reported that anti-Jo-1 Abs are closely associated with myositis [14], [17], whereas patients with anti-KS are more likely to have ILD without clinical evidence of myositis [18], [23]. On the other hand, Sato *et al* previously reported that the presence of anti-PL-7 is closely associated with PM/DM-SSc overlap as well as ILD in Japanese patients [24].

This is a large comprehensive study to focus on the clinical and laboratory features in adult patients with anti-ARS Abs for the investigation of similarities and differences in these anti-ARS Abs. The results of this study indicate that anti-ARS Abs share several clinical features, but also have some considerable differences. Thus, identification of each anti-ARS Ab is beneficial to define this rather homogeneous subset of patients and to predict clinical outcomes.

## Patients and Methods

### Ethics Statement

Ethical approval for the study was obtained from the individual institutional review boards (Kanazawa University, Keio University, Nagasaki University, St. Marianna University, Social Insurance Chukyo Hospital, and Ogaki Municipal Hospital) and all sera were collected after the subjects gave their written informed consent.

### Patients and Sera

Serum samples were obtained from Japanese patients with autoimmune diseases or related disorders who had visited Kanazawa University Hospital or collaborating medical centers from 2003 to 2009. In total, 3164 samples (from 478 patients with DM/PM, 498 with SSc, 183 with ILD alone, 376 with SLE, 102 with mixed connective tissue disease, 398 with Sjogren’s syndrome, and 1129 with rheumatoid arthritis) were screened by immunoprecipitation (IP) assay for the detection of antinuclear or anticytoplasmic antibodies. These patients were referred mainly by rheumatologists, dermatologists, or pulmonologists. PM and classic DM were defined by fulfillment of the Bohan and Peter criteria for definite or probable diagnoses [25]. DM was distinguished from PM based on the presence of heliotrope rash or Gottron’s lesions (Gottron’s papules and/or Gottron’s sign). The diagnosis of CADM was based on the criteria proposed by Sontheimer [26], as follows: clinical skin manifestations typical of DM but minimal or no clinical features of myositis for >2 years after the onset of skin manifestations. All patients with SLE or SSc fulfilled the American College of Rheumatology criteria [27], [28]. PM/DM-overlap was diagnosed by the coexistence of SLE and/or SSc in addition to PM or DM. “ILD alone” was defined by the presence of ILD without fulfillment of any of the criteria for PM, DM, CADM, SLE, or SSc. Patients with ILD alone were examined for potential coexistence of myositis by evaluating muscle weakness and serum muscle enzyme levels including creatine kinase (CK) and aldolase during follow-up, while those without ILD were examined for potential coexistence of ILD by examining dyspneic symptoms and chest radiograph and/or high-resolution computed tomography (HRCT) at every 3 to 6 months.

Clinical information was collected retrospectively for all patients with anti-ARS Abs by reviewing their clinical charts. Initial manifestations were defined as the clinical presentation at the first clinic visit. Patients who had at least one of the following symptoms: symmetrical proximal muscle weakness, muscle pain, or elevated levels of myogenic enzymes, underwent electromyogram, MRI, and/or muscle biopsy for confirmation of the presence of myositis. Patients were diagnosed with myositis if at least one of these confirmatory examinations showed findings compatible with inflammatory myopathy: a myogenic pattern on electromyogram [25], muscular edema on T2-weighted images with fat suppression on MRI [29], or necrosis, regeneration, and some atrophy of muscle fibers and inflammatory cell infiltration on muscle biopsy [25]. Patients were diagnosed as having ILD according to the images on chest HRCT. RP-ILD was defined as progressive dyspnea and progressive hypoxemia with a worsening of interstitial changes on the chest images within 1 month from the onset of respiratory manifestations [11]. Internal and hematologic malignancies in anti-ARS-positive patients was defined if the malignant disease was diagnosed concurrently with or within 3 years after diagnosis of anti-synthetase syndrome or if a preceding malignant disease occurred within 3 years before diagnosis of anti-synthetase syndrome [4]. Sjögren’s syndrome was defined in accordance with the revised European criteria [30].

### IP Assays

Protein IP assays were carried out with extracts of the leukemia cell line, K562 [11]. A total of 10 µl of the patient’s serum was bound to 2 mg protein-A Sepharose beads (Amersham Biosciences, Piscataway, NJ) in 500 µl of IP buffer (10 mM Tris-HCl, pH 8.0, 50 mM NaCl, 0.1% Nonidet P-40), incubated for 2 h at 4°C, and then washed five times with IP buffer. Ab-coated Sepharose beads were mixed with 100 µl ^35^S-methionine-labelled K562 cell extracts derived from 10^6^ cells and rotated at 4°C for 2 h. After five washes, the beads were resuspended in sodium dodecyl sulphate (SDS) sample buffer and the polypeptides were fractionated by 7.5% SDS-polyacrylamide gel electrophoresis (PAGE) followed by autoradiography. For the analysis of RNA, immunoprecipitated RNA was detected in 8% urea-PAGE from a cell extract obtained from 3×10^6^ non-radiolabeled K562 cells by phenol/chloroform, visualized by silver staining [31]. Each anti-ARS Ab was considered positive if serum samples produced precipitin lines with immunological identity to reference sera by both protein and RNA IP [32]. Anti-Ro Ab and anti-La Ab were detected by IP assays as well. Serum was considered positive for anti-Ro Ab if at least one of the Y1–Y5 RNAs was detected by RNA IP and the 60 kDa protein was detected by protein IP; serum was considered positive for anti-La Ab if RNAs contained in the 7S and 5.8S lesions were detected by RNA IP and the 48 kDa protein was detected by protein IP.

### Immunofluorescence

Indirect immunofluorescence tests were carried out with slides of monolayer HEp-2 cells (Medical & Biological Laboratories [MBL], Nagoya, Japan) as substrate [33]. Anticentromere antibody was considered positive if serum diluted at 1∶40 produced a characteristic staining pattern on HEp-2 cells as well as on commercially prepared HeLa cell chromosomal spreads (MBL) [34].

### Statistical Analysis

Frequencies among all six anti-ARS-positive subgroups were compared with a chi-square test. If the overall P value was less than 0.05, pairwise comparisons were performed with a chi-square test with Yates’ correction where appropriate. Continuous variables confirmed to be normally distributed were shown as mean and SD, and their comparisons among groups were carried out with an ANOVA. All statistical analyses were performed with StatView software.

## Results

### Detection of Anti-ARS Abs

**Figure 1:**
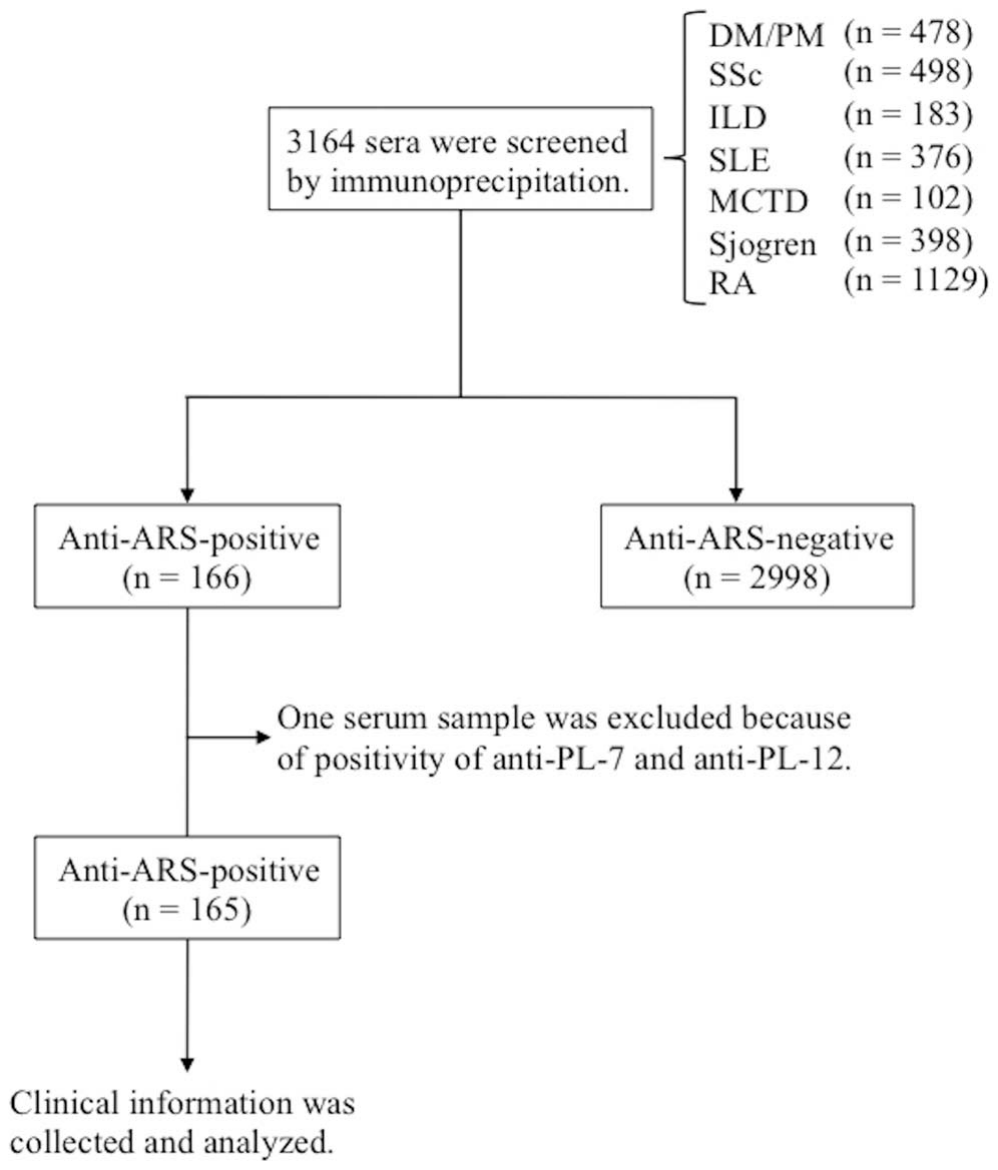
Enrollment and selection of patients. DM; dermatomyositis, PM; polymyositis, SSc; systemic sclerosis, ILD; interstitial lung disease, SLE; systemic lupus erythematosus, MCTD; mixed connective tissue disease, Sjogren; Sjogren’s syndrome, RA; rheumatoid arthritis.

**Figure 2:**
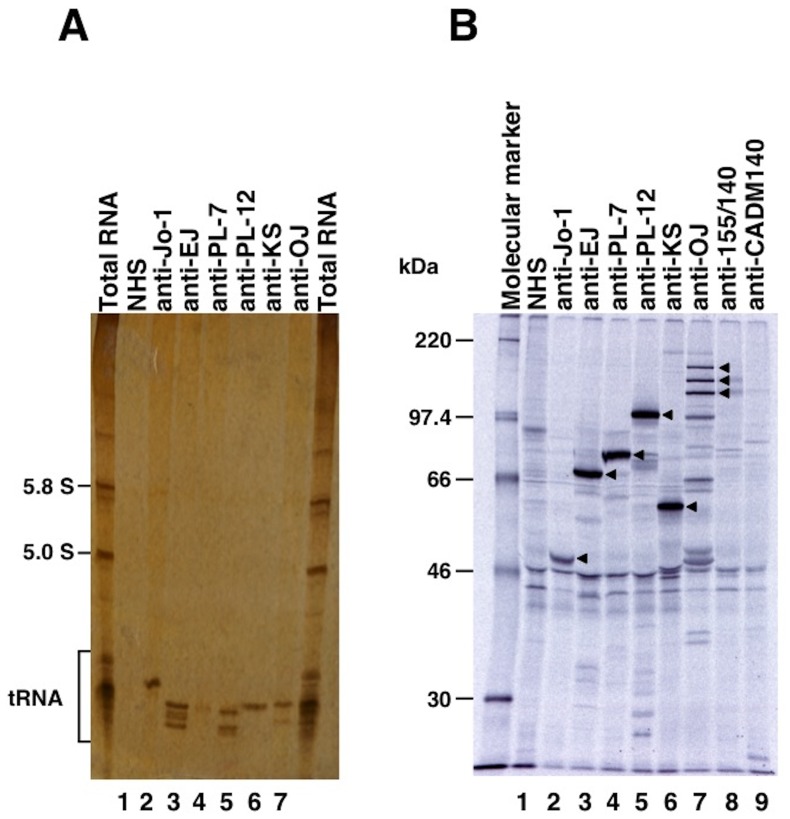
Representative immunoprecipitation assay for RNA with anti-aminoacyl-tRNA synthetase (anti-ARS) sera. **A,** Immunoprecipitation of histidyl-tRNA synthetase, glycyl-tRNA synthetase, threonyl-tRNA synthetase, alanyl-tRNA synthetase, asparaginyl-tRNA, and isoleucyl-tRNA synthetase by sera. K562 cell extracts were immunoprecipitated with sera, and RNA was extracted, electrophoresed on 8% urea-polyacrylamide gels, and visualized by silver staining. Total RNA, with the 5.8 and 5.0 S small ribosomal RNAs and the tRNA region indicated; Lane 1, normal health serum (NHS) indicated; Lanes 2–7: anti-ARS sera indicated, with antibodies to Jo-1 (histidyl-tRNA synthetase), EJ (glycyl-tRNA synthetase), PL-7 (threonyl-tRNA synthetase), PL-12 (alanyl-tRNA synthetase), KS (asparaginyl-tRNA synthetase), and OJ (isoleucyl-tRNA synthetase). **B**, Immunoprecipitation of ^35^S-methionine-labeled K562 cell extracts was performed on anti-ARS sera and NHS, separated on 10% SDS-PAGE, and analyzed by autoradiography. Molecular weight markers include protein bands corresponding to 220, 97.4, 66, 46, and 30 kDa.

Of 3164 samples screened by IP assays, anti-ARS Abs were detected in 166 patients (5.2%) (Figure 1). As shown in Figure 2, 6 anti-ARS specificities, including anti-Jo-1, anti-EJ, anti-PL-7, anti-PL-12, anti-KS, anti-OJ, were easily detectable and distinguishable by IP assays. Of 166 patients with anti-ARS Abs, anti-Jo-1 was found in 59 (36%) patients, anti-EJ was found in 38 (23%) patients, anti-PL-7 was found in 30 (18%) patients, anti-PL-12 was found in 19 (11%) patients, anti-KS was found in 13 (8%) patients, and anti-OJ was found in 8 (5%) patients. One patient with classic DM had antibodies reactive to both PL-7 and PL-12, and was excluded from the following analyses for clinical associations.

Coexistence of anti-ARS Abs and other autoimmune connective tissue disease-related Abs was examined (Table 1). Antibodies against Mi-2, 155/140, CADM-140/MDA5, MJ/NXP-2, topoisomerase I, centromere, U1RNP, Th/To, U3RNP, Sm and La/SS-B were rarely found in patients with anti-ARS Abs. In contrast, anti-Ro/SS-A Abs were found in 31 (19%) patients. These results were principally consistent with previous findings that myositis-specific Abs are relatively mutually exclusive, while myositis-associated Abs coexist with myositis-specific Abs [13], [35].

**Table 1:** Coexistence of other autoantibodies in patients with anti-aminoacyl-tRNA synthetase antibodies.*

	Anti-Jo-1(n = 59)	Anti-EJ(n = 38)	Anti-PL-7(n = 29)	Anti-PL-12(n = 18)	Anti-KS(n = 13)	Anti-OJ(n = 8)	Anti-PL-7/PL-12 (n = 1)
Anti-Mi-2	0	0	0	0	0	0	0
Anti-155/140	0	0	0	0	0	0	0
Anti-CADM-140/MDA5	0	0	0	0	0	0	0
Anti-MJ/NXP-2	0	0	0	0	0	0	0
Anti-topoisomerase I	0	1	0	0	0	0	0
Anti-centromere	1	0	0	1	2	0	0
Anti-U1RNP	0	0	1	1	0	0	0
Anti-Th/To	0	0	0	1	0	0	0
Anti-U3RNP	1	0	0	0	0	0	0
Anti-Sm	0	0	1	0	0	0	0
Anti-Ro/SS-A	9	9	8	4	1	0	0
Anti-La/SS-B	0	2	2	0	0	0	0

* Values are the number of patients.

### Associations between Clinical Diagnoses and Anti-ARS Abs

**Figure 3:**
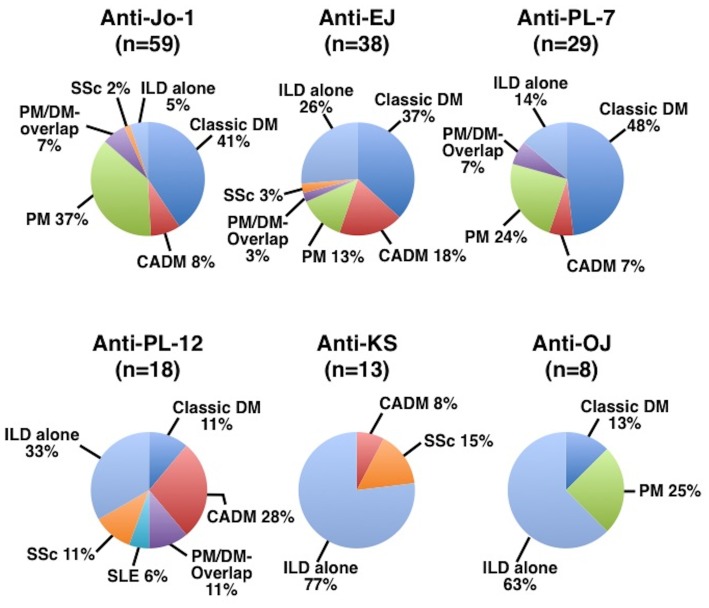
Prevalence of dermatomysitis (DM), clinically amyopathic DM (CADM), polymyositis (PM), PM/DM-overlap, systemic lupus erythematosus (SLE), systemic sclerosis (SSc), and interstitial lung disease (ILD) alone, in each subgroup of anti-synthetase syndrome.

The distributions of classic DM, CADM, PM, PM/DM-overlap, SLE, SSc, and ILD alone in patients with individual anti-ARS Abs are shown in Figure 3. More than half of the patients with anti-Jo-1, anti-EJ, or anti-PL-7 had apparent myositis, including classic DM, PM, and PM/DM-overlap. The proportion with ILD alone was different among patients with various anti-ARS Abs. In particular, 10 of 13 (77%) patients with anti-KS and 5 of 8 (63%) patients with anti-OJ were diagnosed with ILD alone. Some patients with anti-ARS Abs were diagnosed with SSc or SLE, but the frequency was relatively low. Thus, most patients with anti-ARS Abs were diagnosed as having classic DM, CADM, PM, PM/DM-overlap, or ILD alone, while the proportion of these diagnoses was different among the subgroups of each anti-ARS Ab.

### Comparison of Clinical Features among Patients with Anti-ARS Abs

A total of 95 patients with anti-ARS Abs had myositis and were diagnosed as having classic DM, PM, or PM/DM-overlap. We first compared clinical features between patients with myositis in the presence and absence of anti-ARS Ab (n = 95 and 152, respectively). Anti-ARS-positive patients with myositis had higher frequencies of Raynaud’s phenomenon (*P* = 0.034), ILD (*P*<0.0001), and polyarthritis (*P* = 0.0015) compared with anti-ARS-negative patients with myositis. There was no difference in the frequency of fever between the two groups (*P* = 0.87).

Then, we compared the demographic features among anti-ARS-based subgroups, as shown in Table 2. No differences were found in age of onset or sex. We next compared muscle weakness and ILD among individual anti-ARS subgroups, both at the initial visit and during the entire follow-up period. Muscle weakness was found in 71 (43%) patients at the initial visit and 95 (58%) during the entire follow-up period, but the frequencies varied among anti-ARS-based subgroups (overall *P* = 0.0011 and *P*<0.0001, respectively). Patients with anti-Jo-1, anti-EJ, and anti-PL-7 had a higher frequency of muscle weakness (59%, 39%, and 52%, respectively, at the initial visit and 78%, 55%, and 76%, respectively during the entire follow-up period) than those with anti-PL-12 (17% for both), anti-KS (7% for both), and anti-OJ (25% for both). In contrast, most patients had ILD at the initial visit, and almost all patients eventually suffered from ILD. While most of them had the chronic type of ILD, a total of 13 patients (8 with anti-Jo-1, 4 with anti-EJ, and 1 with anti-PL-7) developed RP-ILD at their first visit or during their clinical course. Thus, the frequency of muscle weakness varied among anti-ARS subgroups, while ILD was observed at equally high frequencies among these subgroups.

**Table 2:** Comparison of clinical features in 165 adult Japanese patients with anti-aminoacyl-tRNA synthetase antibodies.*

	Anti-Jo-1(n = 59)	Anti-EJ(n = 38)	Anti-PL-7(n = 29)	Anti-PL-12(n = 18)	Anti-KS(n = 13)	Anti-OJ(n = 8)	Overall*P*
Age at onset, median (range), y	53 (22–76)	53 (18–78)	53 (25–79)	48 (20–75)	54 (39–67)	57 (32–79)	0.61
No. of females/no. of males	43/16	32/6	26/3	16/2	7/6	6/2	0.077
Clinical features (at initial visit)							
Interstitial lung disease	71	84	76	89	100	100	0.077
Muscle weakness	59	39	52	17	7	25	0.0011^a^
Clinical features (entire follow-up period)							
Fever	27	39	34	44	8	13	0.16
Raynaud’s phenomenon	19	13	38	44	31	13	0.044^b^
Interstitial lung disease	90	97	93	94	100	100	0.56
Muscle weakness	78	55	76	17	7	25	<0.0001^c^
Polyarthritis	58	24	31	22	31	13	0.0029^d^
Erosive arthritis	12	5	0	17	23	0	0.16
Malignancy	15	3	7	17	15	25	0.22
Sjögren’s syndrome	7	16	14	0	8	0	0.32
Skin manifestations							
Heliotrope rash	7	21	38	17	0	0	0.0019^e^
Gottron’s sign (hand)	44	45	41	33	8	13	0.10
Gottron’s sign (elbow and/or knee)	27	39	45	33	0	13	0.043^f^
Mechanic’s hands	56	29	45	22	23	38	0.031^g^
Laboratory findings							
CK, IU/L, mean ± SD	2213±3168	1681±2967	1768±2096	250±306	143±84	881±1129	0.024^h^
LDH, IU/L, mean ± SD	595±5961	427±223	565±406	346±187	215±77	355±197	0.019^i^
KL-6, U/mL, mean ± SD	1335±2067 (n = 54)	1425±1030	1374±1444	1630±1650	1527±1404 (n = 12)	1307±877	0.99
SP-D, ng/mL, mean ± SD	206±229 (n = 39)	318±626 (n = 36)	229±275 (n = 25)	250±170 (n = 15)	185±129	123±53 (n = 6)	0.74

* Unless noted otherwise, values are percentages of patients. NS: not significant; CK: creatine kinase; LDH: lactate dehydrogenase. One patient with DM who had antibodies reactive to both PL-7 and PL-12 was excluded from the analysis. Significant differences (overall *P*<0.05) were further analyzed by pairwise comparisons.

a *P*<0.05 between anti-PL-7 and anti-PL-12; *P*<0.01 between anti-Jo-1 and anti-PL-12, and between anti-KS and anti-Jo-1 or anti-PL-7;

b *P*<0.05 between anti-Jo-1 and anti-PL-7 or anti-PL-12, and between anti-EJ and anti-PL-7; *P*<0.01 between anti-EJ and anti-PL-12.

c *P*<0.05 between anti-EJ and anti-PL-12; *P*<0.01 between anti-Jo-1 and anti-PL-12, anti-KS or anti-OJ, between anti-EJ and anti-KS, and between anti-PL-7 and anti-PL-12, anti-KS or anti-OJ.

d *P*<0.05 between anti-Jo-1 and anti-PL-7, anti-KS or anti-OJ; *P*<0.01 between anti-Jo-1 and anti-EJ or anti-PL-12.

e *P*<0.05 between anti-Jo-1 and anti-EJ; *P*<0.01 between anti-PL-7 and anti-Jo-1 or anti-KS.

f *P*<0.05 between anti-KS and anti-EJ or anti-PL-12; *P*<0.01 between anti-PL-7 and anti-KS.

g *P*<0.05 between anti-Jo-1 and anti-PL-12 or anti-KS; *P*<0.01 between anti-Jo-1 and anti-EJ.

h *P*<0.05 between anti-EJ and anti-PL-12 or anti-KS; *P*<0.01 between anti-Jo-1 and anti-PL-12 or anti-KS, and between anti-PL-7 and anti-PL-12 or anti-KS.

i *P*<0.05 between anti-PL-7 and anti-PL-12; *P*<0.01 between anti-Jo-1 and anti-PL-12, and between anti-KS and anti-Jo-1, anti-EJ or anti-PL-7.

Fever, Raynaud’s phenomenon, polyarthritis, and mechanic’s hands during the entire follow-up period were compared among anti-ARS subgroups. The frequency of fever varied among anti-ARS-based subgroups (8–44%), but there was no statistical difference. Raynaud’s phenomenon was found in 40 of 165 (24%) patients with anti-ARS Abs and more frequently observed in patients with anti-PL-12 and anti-PL-7 (overall *P* = 0.044). Polyarthritis was most common in patients with anti-Jo-1 (58%) and infrequently observed in patients with anti-OJ (13%) (overall *P* = 0.0029). Mechanic’s hands, which are the representative skin manifestation in anti-synthetase syndrome, were observed in all anti-ARS Ab-based subgroups, but the frequency was highest in patients with anti-Jo-1 (56%) (overall *P* = 0.031). Collectively, Raynaud’s phenomenon, polyarthritis, and mechanic’s hands were observed in each anti-ARS Ab subgroup, but the frequencies were rather heterogeneous.

We then compared heliotrope rash and Gottron’s signs, which are the representative skin manifestations in DM. Heliotrope rash was found in 26 of 165 (16%) patients with anti-ARS Abs (overall *P* = 0.0019) and Gottron’s sign (elbow and/or knee) was found in 51 (31%) (overall *P* = 0.043). These manifestations were predominantly found in patients with anti-EJ, anti-PL-7, and anti-PL-12.

With regard to laboratory findings, CK levels were lower in patients with anti-PL-12 and anti-KS (overall *P* = 0.024), and lactate dehydrogenase (LDH) was lowest in patients with anti-KS (overall *P* = 0.019). It is likely that these results were associated with the frequencies of muscle involvement. KL-6 and pulmonary surfactant protein D (SP-D) levels are associated with the activity and severity of ILD [36], [37]. While elevations of both KL-6 and SP-D were observed in all anti-ARS-based subgroups, no significant differences were observed in serum KL-6 and SP-D levels.

As an association of malignancy with PM/DM has been reported, we examined the frequency of malignancies in patients with anti-ARS Abs (Table 2). Malignancies were observed in 19 (12%) of 165 patients with anti-ARS Abs, and 1 of those had a double malignancy. A summary of the malignancies is listed in Table 3. There were 4 patients with colon cancer, 4 with gastric cancer or carcinoid, 3 with breast cancer, 3 with lung cancer, and single cases of prostate cancer, nasopharyngeal cancer, uterine corpus cancer, thyroid cancer, ovarian cancer, and non-Hodgkin lymphoma. There was no trend in the prevalence of malignancy or the type of malignancy among anti-ARS-based subgroups. Seven of 19 patients with malignancy simultaneously developed PM/DM or ILD, while 7 of 19 had malignancy prior to the development of PM/DM or ILD, and 5 of 19 developed malignancy after the diagnosis of PM/DM or ILD.

**Table 3:** Summary of malignancy in patients with anti-aminoacyl-tRNA synthetase antibodies.

Anti-ARS	Age, y	Sex	Diagnosis	ILD	Type of malignancy	Onset
Anti-Jo-1	54	M	PM	−	Lung cancer	At same time
Anti-Jo-1	59	F	DM	+	Gastric cancer	Before DM
Anti-Jo-1	38	F	DM	+	Ovarian cancer	At same time
Anti-Jo-1	54	M	PM	+	Colon cancer	After PM
Anti-Jo-1	74	M	DM	+	Colon cancer	Before DM
Anti-Jo-1	42	F	DM	+	Breast cancer	Before DM
Anti-Jo-1	67	F	DM	+	Non-Hodgkinlymphoma	At same time
Anti-Jo-1	62	M	PM	−	Gastric cancer	After PM
Anti-Jo-1	57	F	DM	+	Thyroid cancer	At same time
Anti-EJ	43	F	DM	+	Nasopharyngeal cancer	At same time
Anti-PL-7	70	F	DM	+	Breast cancer	Before DM
Anti-PL-7	79	M	ILD	+	Gastric cancer	After ILD
Anti-PL-12	53	F	ILD	+	Lung+uterinecorpus cancer	Before ILD
Anti-PL-12	66	M	ILD	+	Colon cancer	After ILD
Anti-PL-12	59	F	DM	+	Breast cancer	Before DM
Anti-KS	59	M	ILD	+	Lung cancer	After ILD
Anti-KS	66	M	ILD	+	Prostate cancer	Before ILD
Anti-OJ	71	F	DM	+	Gastric carcinoid	At same time
Anti-OJ	77	M	PM	+	Colon cancer	At same time

ILD: interstitial lung disease; PM: polymyositis; DM: dermatomyositis.

### Causes of Death

Sixteen (10%) of 165 anti-ARS-positive patients died during the follow-up period (Table 4). Causes of death included ILD in 8, malignancy in 3, infection in 2, and one each of myocardial infarction, rupture of an abdominal aortic aneurysm, and hypertrophic cardiomyopathy.

**Table 4:** Cause of death in patients with anti-aminoacyl-tRNA synthetase antibodies.

Anti-ARS	Age, y	Sex	Diagnosis	ILD	Cause of death	Time after diagnosis (y)
Anti-Jo-1	64	F	DM	+	ILD	0.3
Anti-Jo-1	38	F	DM	+	Infection	3
Anti-Jo-1	36	F	DM	+	ILD	5.5
Anti-Jo-1	62	M	PM	−	Gastric cancer	5
Anti-EJ	65	F	DM	+	ILD	2.5
Anti-EJ	55	F	ILD	+	ILD	0.6
Anti-EJ	55	F	DM	+	ILD	4.25
Anti-EJ	53	F	SSc	+	Infection	6
Anti-EJ	50	F	DM	+	Myocardial infarction	5.25
Anti-PL-7	63	F	DM	+	ILD	1.8
Anti-PL-7	71	F	DM	+	ILD	3
Anti-PL-7	75	M	ILD	+	ILD	0.3
Anti-PL-12	53	F	ILD	+	Lung cancer	3
Anti-PL-12	74	F	DM	+	Rupture of an abdominal aortic aneurysm	0.6
Anti-PL-12	75	F	ILD	+	Hypertrophic cardiomyopathy	2
Anti-KS	59	M	ILD	+	Lung cancer	1.5

ILD: interstitial lung disease; DM: dermatomyositis; PM: polymyositis; SSc: systemic sclerosis.

### Timing of Development of ILD and Myositis in Patients with Anti-ARS Abs

**Figure 4:**
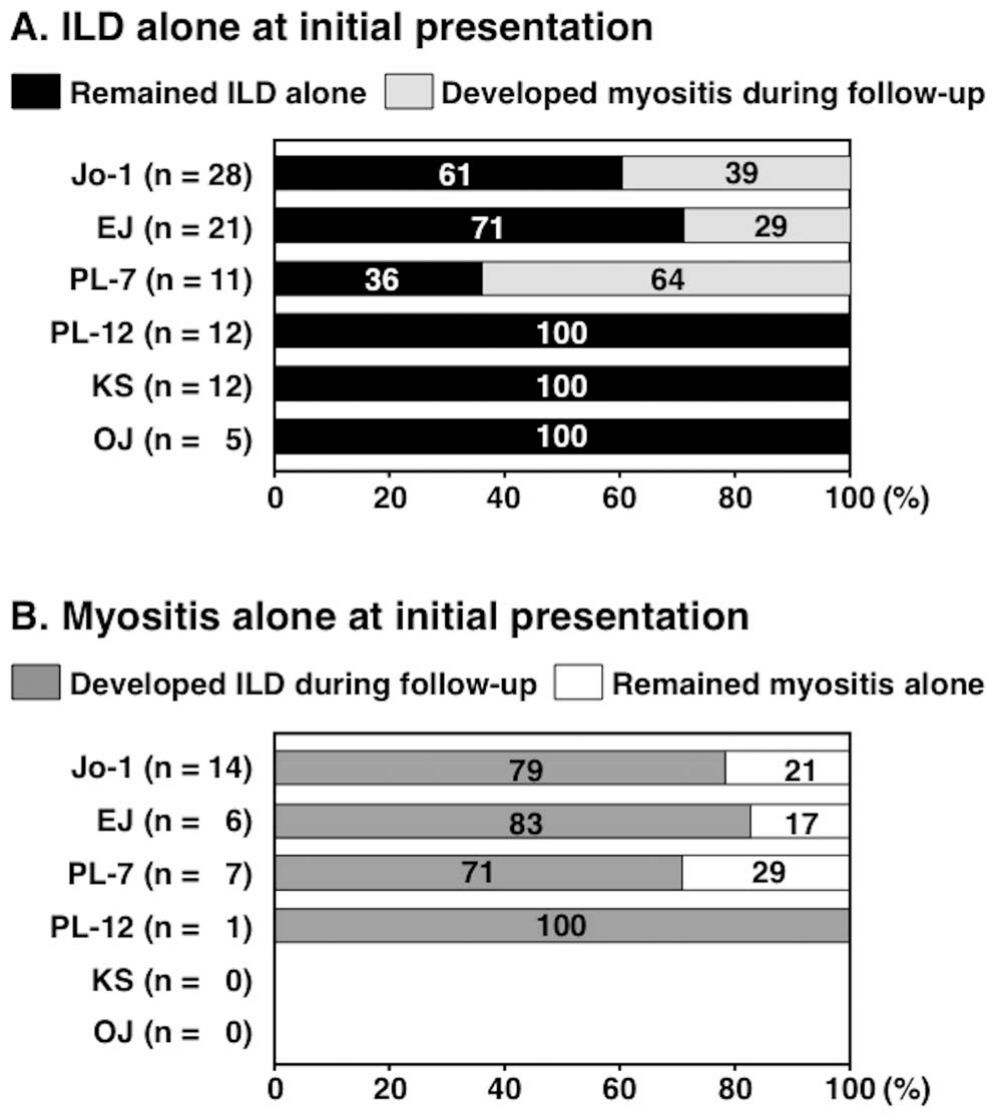
The clinical course of anti-synthetase syndrome patients who developed myositis or interstitial lung disease (ILD) with or without skin manifestations at disease onset. According to the clinical course, patients were classified into four types: remained with ILD alone, developed myositis during follow-up, developed ILD during follow-up, and remained with myositis alone. The clinical course of those who had ILD with or without skin manifestations, but without muscle involvement at their first assessment (A), and the clinical course of those who had myositis with or without skin manifestations, but without ILD at their first assessment (B).

Initial manifestations in patients with anti-ARS Abs are summarized in Table 5. At initial presentation, the combination of manifestations, including DM rashes, myositis, and ILD, varied among patients with anti-ARS Abs. The frequency of ILD alone at presentation was different among groups stratified by anti-ARS Abs (overall *P* = 0.0001). While some patients with anti-ARS Abs had 2 or more manifestations at initial diagnosis, others sequentially developed different manifestations, even when they were receiving therapy. Thus, we analyzed the timing of development of ILD and myositis. Figure 4A includes patients with ILD alone and DM rashes and ILD, and Figure 4B includes those with myositis alone and DM rashes and myositis at initial presentation. Patients with DM rashes alone, myositis and ILD, DM rashes, myositis, and ILD, and none of DM rashes, myositis, and ILD were excluded from this analysis. We assessed whether patients who had ILD alone at presentation developed myositis during follow-up (Figure 4A). As a result, 39%, 29%, and 64% of patients with anti-Jo-1, anti-EJ, and anti-PL-7, respectively, subsequently developed myositis. In contrast, none of the patients with anti-PL-12, anti-KS, and anti-OJ who had ILD alone at presentation developed myositis later in the course of the disease. The distribution of the frequencies for developing myositis among anti-ARS-based subgroups was statistically significant (overall *P* = 0.0008). In contrast, when patients who had myositis without ILD at presentation were selected, nearly all of them developed ILD later in the course of the disease (Figure 4B). There was no difference in observation period among the 6 groups (Jo-1, 62±24; EJ, 56±27; PL-7, 50±27; PL-12, 53±27; KS, 70±20; and OJ, 62±32 months). In addition, there was no difference in initial treatment regimen among the 6 groups stratified by anti-ARS Abs (Table 6), although 38% of patients with anti-KS did not receive immunosuppressive therapy and this frequency was highest among the 6 groups (overall *P* = 0.0070). Almost all patients with anti-ARS Abs who had ILD or myositis received immunosuppressive treatment, including corticosteroids alone or in combination with immunosuppressants. Accordingly, patients with anti-PL-12, anti-KS, or anti-OJ were less likely to develop myositis during follow-up than those with anti-Jo-1, anti-EJ, or anti-PL-7.

**Table 5:** Initial manifestations in patients with anti-aminoacyl-tRNA synthetase antibodies.*

	Anti-Jo-1(n = 59)	Anti-EJ(n = 38)	Anti-PL-7(n = 29)	Anti-PL-12(n = 18)	Anti-KS(n = 13)	Anti-OJ(n = 8)	Overall*P*
DM rashes alone	2	0	14	11	8	0	0.14
Myositis alone	14	11	21	0	0	0	0.14
ILD alone	29	39	28	56	92	63	0.0001^a^
DM rashes and Myositis	10	5	4	6	0	0	0.45
DM rashes and ILD	19	16	10	11	0	0	0.46
Myositis and ILD	7	13	7	0	0	25	0.24
DM rashes, Myositis, and ILD	10	16	17	11	0	13	0.75
No DM rashes, Myositis, or ILD**	10	0	0	6	0	0	0.11

* Values are percentages of patients.

** These patients had polyarthritis at presentation. Significant differences (overall *P*<0.05) were further analyzed by pairwise comparisons.

a *P*<0.05 between anti-PL-12 and anti-Jo-1 or anti-KS; *P*<0.01 between anti-KS and anti-Jo-1, anti-EJ or anti-PL-7.

**Table 6:** Initial treatment in patients with anti-aminoacyltransfer RNA synthetase antibodies.*

	Anti-Jo-1(n = 59)	Anti-EJ(n = 38)	Anti-PL-7(n = 29)	Anti-PL-12(n = 18)	Anti-KS(n = 13)	Anti-OJ(n = 8)	Overall*P*
No immunosuppressive therapy	7 (4)	5 (2)	3 (1)	11 (2)	38 (5)	13 (1)	0.0070^a^
Initial treatment							
CS oral only	68 (40)	68 (26)	59 (17)	67 (12)	46 (6)	88 (7)	0.45
CS pulse+oral	8 (5)	16 (6)	21 (6)	6 (1)	8 (1)	0 (0)	0.36
CS (pulse and/or oral)+CsA	10 (6)	3 (1)	3 (1)	11 (2)	0 (0)	0 (0)	0.41
CS (pulse and/or oral)+Tac	2 (1)	0 (0)	3 (1)	0 (0)	0 (0)	0 (0)	0.81
CS (pulse and/or oral)+CY(oral and/or iv)	3 (3)	0 (1)	0 (0)	0 (0)	0 (0)	0 (0)	0.82
CS (pulse and/or oral)+CsA orTac+CY (oral and/or iv)	0 (0)	0 (0)	7 (2)	6 (1)	0 (0)	0 (0)	0.17
CS (pulse and/or oral)+MZR	0 (0)	3 (1)	3 (1)	0 (0)	0 (0)	0 (0)	0.69
CS (pulse and/or oral)+Buc	0 (0)	3 (1)	0 (0)	0 (0)	8 (1)	0 (0)	0.25

* Values are percentages of patients. Patient numbers are given in parenthesis. CS: corticosteroid; CsA: cyclosporine A; Tac: tacrolimus; CY: cyclophosphamide; iv: intravenous administration; MZR: mizoribine; Buc: bucillamine. Significant differences (overall *P*<0.05) were further analyzed by pairwise comparisons.

a *P*<0.01 between anti-KS and anti-Jo-1, anti-EJ or anti-PL-7.

## Discussion

This comprehensive report aimed to compare clinical features among anti-ARS-based subgroups on a large scale. As reported previously, more than one anti-ARS Ab did not coexist in general. While this study confirmed that ILD, myositis, Raynaud’s phenomenon, polyarthritis, and mechanic’s hands were common manifestations in anti-synthetase syndrome, the frequencies of each manifestation varied. That is, myositis was well associated with anti-Jo-1, anti-EJ, and anti-PL-7. Additionally, a substantial number of patients positive for anti-EJ or anti-PL-12 had CADM. Therefore, most of the clinical diagnoses were PM or DM for anti-Jo-1, anti-EJ, and anti-PL-7; CADM or ILD for anti-PL-12; and ILD for anti-KS and anti-OJ. Although patients with anti-ARS Abs share several common manifestations, it is likely that each of these Abs defines a clinically distinct phenotype and may serve as a predictor for clinical complications.

Since nearly all patients with anti-ARS Abs had ILD, this study confirms previous findings that anti-ARS Abs are a marker for ILD [38]–[42]. Most of the clinical diagnoses in patients with anti-ARS Abs were classic DM, CADM, PM or ILD alone in this study. This finding was also in accordance with previous reports that anti-ARS Abs were highly specific for a proportion of patients with PM, DM, or ILD [4], [38], [43]–[45]. However, classic DM, CADM, or PM was found predominantly in patient subgroups with anti-Jo-1, anti-EJ, and anti-PL-7, whereas two-thirds of patients with anti-PL-12 were diagnosed with CADM or ILD. In contrast, anti-KS and anti-OJ were associated with ILD alone. Therefore, it is likely that the clinical diagnosis varies among anti-ARS-based subgroups.

Regarding myositis, it appears that anti-ARS Abs are divided into myositis-related and non-myositis-related subgroups. Anti-Jo-1, anti-EJ, and anti-PL-7 belong to the myositis-related subgroup, since myositis was found in at least half of the patients with these anti-ARS Abs. These findings agreed with previous reports describing a relationship of myositis with anit-Jo-1 [46], anti-EJ [13], [17], [47], [48], and anti-PL-7 [24], [49]. In contrast, anti-PL-12, anti-KS, and anti-OJ were not well related to myositis in this study. These results also paralleled those of former reports that anti-KS is highly associated with ILD [32], [48]. However, rates of myositis in anti-PL-12 and anti-OJ appear to be different from previous reports. Of a total of 47 cases with anti-PL-12, muscle weakness was observed in 27 (57%) patients [16], [23], [50]. Sato *et al* reported 7 Japanese patients with anti-OJ, in which muscle weakness was seen in 4 patients [51]. Thus, whether anti-PL-12 and anti-OJ are related to myositis remains controversial. Collectively, patients with anti-ARS Abs form a basically homogenous clinical entity, as previously reported; mutual comparisons in this study elucidated certain differences in clinical features among patients with specific anti-ARS Abs.

Regarding skin manifestations, this study revealed an interesting observation. The main clinical diagnoses in anti-Jo-1, anti-EJ, anti-PL-7, and anti-PL-12 were classic DM or CADM. This resulted from the higher frequencies of DM-specific skin manifestations in these patients, which included heliotrope rash and Gottron’s signs. However, the distribution of skin manifestations varied among anti-ARS Abs. Only less than 10% of patients with anti-Jo-1 had heliotrope rash, while approximately 20–30% of those with anti-EJ, anti-PL-7, and anti-PL12 had this eruption. On the other hand, the frequency of anti-Jo-1-positive patients who had Gottron’s sign was similar compared to those with anti-EJ, anti-PL-7, and anti-PL-12. Thus, the prevalence of DM-specific skin manifestations is not identical among different anti-ARS Abs, even though the main diagnosis is classic DM or CADM.

With respect to the onset of evident manifestations of myositis and ILD, these patients were divided into three groups: i) patients with myositis preceding ILD; ii) patients with ILD preceding myositis; and iii) patients with simultaneous onset of both conditions. We reported previously that the onset of anti-synthetase syndrome is acute, but that the development of myositis may lag behind the onset of ILD in anti-ARS-positive DM patients [38]. A similar finding was described in another report [44]. In this study, most patients with anti-ARS Abs who had myositis without ILD at the onset of disease developed ILD later. On the other hand, the rate of subsequent occurrence of myositis differed among the subsets of anti-ARS Abs when the patients had ILD without myositis as their initial manifestation. Thus, screening and identification of anti-ARS Abs is found to be beneficial in predicting the onset of ILD.

Other than ILD and myositis, previous reports described that arthritis, Raynaud’s phenomenon, fever, and mechanic’s hands are common clinical features in anti-synthetase syndrome [21], [40], [44]. There was no significant difference in the frequency of fever in this study. On the other hand, this study revealed some differences in the frequencies of polyarthritis, Raynaud’s phenomenon, and mechanic’s hands. While these three manifestations were observed with each anti-ARS Ab at a comparable rate, polyarthritis and mechanic’s hands were most frequently found with anti-Jo-1, and Raynaud’s phenomenon was most frequently found with anti-PL-12. Nonetheless, the differences in frequencies of these manifestations among anti-ARS subgroups were less evident than that with myositis.

We acknowledge several limitations of this study. First, it included a relatively small number of patients with anti-PL-12, anti-KS, or anti-OJ. Second, most facilities enrolled in this study were referral centers. This study had a higher frequency of DM and a relatively lower frequency of PM compared with other similar studies. This may be explained by the fact that our patients were mainly referred to us by rheumatologists, dermatologists, and pulmonologists, and only a few of them were referred by neurologists. Therefore, we cannot exclude selection bias. Third, the possibility cannot be ruled out that coexistence of anti-Ro/SS-A Abs influence the clinical feature of anti-ARS-positive patients with anti-Ro/SS-A Abs, as anti-Ro/SS-A Abs are considered as myositis-associated Abs and form the subgroup. In the analysis of clinical course, possibilities are raised that the short observation period and the differences in treatment potentially affected the results. Additionally, patients who visited to referral centers were examined for the existence of myositis and they were categorized by Bohan and Peter and Sontheimer criteria that are commonly used for diagnosis of myositis in a current condition. However, as clinical features of patients with anti-ARS Abs are largely heterogeneous, it appears difficult to stratify the patients by current criteria. It may be clinically useful to classify the anti-ARS-positive patients based on the type of anti-ARS Abs, not current criteria. It needs to consider the conformity of the classification of the patients with anti-ARS Abs with diagnosis criteria for myositis. Indeed, Connors *et al* have proposed the criteria for anti-ARS syndrome as follows [40]. First, patients must have positive serologic testing for anti-ARS Abs. Then, patients have one or more of the following conditions: Evidence of myositis by Bohan and Peter criteria, evidence of ILD by American Thoracic Society criteria, evidence of arthritis by clinical examination, radiographic findings, or patient self-report, unexplained, persistent fever, Raynaud’s phenomenon, and mechanic’s hands. Therefore, more studies are needed for a better general understanding of the clinical characteristics of patients with anti-ARS Abs.

In summary, although anti-ARS Abs share common clinical features, each anti-ARS Ab appears to form some distinct clinical subset. However, the identification of anti-ARS Abs (except for anti-Jo-1) is limited only to certain facilities, as it requires a complicated technique. Establishment of a system routinely available to screen all anti-ARS Abs specificities is needed.
